# Monthly Summary

**Published:** 1889-05

**Authors:** 


### Monthly Summary.

WASHING AMALGAM.—*By Thomas Fletcher, F. C. S.*—Dr. Ingersoll seems to be very much mixed on the question of mixing amalgams. There is a serious difference between mixing a quick setting alloy with a small quantity of mercury and packing this at once under water, and doing as Dr. Ingersoll does, *i. e.*, flooding the fillings with mercury and squeezing it out and washing. In the first case we get an alloy which contains a very small quantity of mercury and which packs hard at once almost like cohesive gold, owing to its quick setting; it is practically unaffected by the trace of moisture present, as Dr. Ingersoll will find if he makes any careful tests for alteration of form. If any excess of mercury is once added, it can never be removed, the alloy is much slower in setting, and is much more liable to be interfered with as regards its form if any moisture whatever is present. He calmly ignores any tests so to stability of form of plugs, and judges solely by the appearance in the hand. Further than this, he makes certain statements as to what is washed away from an amalgam without the slightest basis for his statements, as he evidently has made no analysis. What he can wash away from an amalgam depends largely on the state of division of the metal, and the chemicals he uses to produce the discoloration which is to be washed away. If Dr. Ingersoll will take the trouble to make a few large plugs in glass tubes, say three-eighths or half-inch diameter, and will learn how to make these permanently water-tight and colour tight for, say, six months, he will not need to trouble his mind about washing or otherwise, he will know all about it so far as his individual working is concerned, and he will know how to produce better amalgam plugs than he apparently does at present.

---

TEETH OF SOME ANIMALS.—B. G. Hays has an interesting article in the *Dental Register* on this subject. Among other things we are told the sloth, armadillo and ant-eater are edentalous, that is, they have no teeth. Some animals have only one, an upper central incisor.

The cetacea, or whale class, have teeth similar in size and appearance, tho in some varieties a network or sive of bone (whale bone) takes the place of teeth. In this class the porpoise has from eighty to ninety teeth, and the dolphin two hundred, all quite similar in size and shape. The sperm whale has but few teeth in the upper jaw, and these are stunted and only partially developed; they are sometimes quite buried in the dense gum during the whole life of the animal. In the lower jaw they are numerous. The norwhal, or North whale, has but a single tooth that is of use, it has one other that is seldom erupted.

The teeth of the rengulata, or hoof animals, vary in number and character. The pig has forty-four.

The hollow horned ruminants, including sheep, cattle and antelopes, with most of the solid horned ruminants, have usually ten teeth below and six above, the upper jaw lacking the incisors and cuspids.

In the camel there is but one incisor above; this antagonizes with three below. It has three back teeth above apposing two below.

Animals with a trunk, as the elephant, which is the only species of his genus extant, has two upper incisors (which prolong into tusks) and a succession of molar teeth which re place each other from behind forward. There are six on each side of each jaw, but only one or parts of two exposed at the same time.

Rodents, or gnawing animals, as the rat, usually have two incisors, tho the hare and the rabbitt, which belong to this class, have four in the upper jaw, The molars vary from three to six.

The carnivorous, or flesh eating animals, have serrated teeth, greatly varying in size and shape, but rather uniform in number. The cat is a pretty good example. It has three incisors, one cuspid, three bicuspids and one molar in the upper jaw, and three incisors, one cuspid, two bicuspids and one molar below.

Men and monkeys have thirty-two teeth, tho the monkeys of the old world are in advance of the men in having an additional bicuspid on each side above and below.

Teeth not only differ in shape and number, but in character. The teeth of the shark, for instance, and some other fish are embedded in the mucous membrane so as to turn back as food is received, and then resuming their upright position, prevent its escape. Other teeth are shed in sections as layers.—*Items of Interest.*

IMPRESSION TAKING.—Modelling composition may be classed next to plaster-of-Paris as an impression material. Fortunately it has qualities which indicate its use where plaster-of-Paris is wanting. Teeth standing at different angles or with large crowns and small necks are taken best with modelling composition.

Now, if we could make a combination of these two materials in obtaining an impression in such a manner that only the best qualities of each would be used, we would obtain much better results than by the universal use of any one material; therefore, I have a method to present which I have been using for sometime with the most satisfactory results. It is especially applicable in partial upper cases.

With a spatula made of a material which can easily be bent in any shape, as of block tin or impression tray material, plaster-of-Paris of the usual consistence for impressions is carried to the roof of the mouth and there spread upon the mucous membrane as far back as its desired to make the plate; more plaster is added to this until it is even full down to the necks of the teeth. The lower surface is to be roughened for a purpose which will appear further on. Water for modelling compound being heated, in the meantime the impression tray is filled with one-half the usual amount of the composition and placed in position against the teeth and plaster core which by this time has become hard. When cooled remove and varnish both parts. When the varnish is dry, oil the plaster core only, as modelling composition separates more nicely when varnished than when oiled. From this procure the cast in the usual way.

The advantages of such a procedure are, first, the plaster and modelling composition are each manipulated in such a manner that the best qualities only are used; second, the plaster being placed in position against the mucous membrane in its softest state is allowed to harden without any pressure as is required when using a tray; and third, the plaster is completely under control, and is allowed to extend no farther back than is necessary, so that gagging is largely prevented.—*Ohio Journal.*

